# Stability of individual differences in sucralose taste preference

**DOI:** 10.1371/journal.pone.0216431

**Published:** 2019-05-14

**Authors:** Sam Z. Bacharach, Donna J. Calu

**Affiliations:** 1 Department of Anatomy and Neurobiology, University of Maryland School of Medicine, Baltimore, Maryland, United States of America; 2 Program in Neuroscience, University of Maryland School of Medicine, Baltimore, Maryland, United States of America; University of Leicester, UNITED KINGDOM

## Abstract

Outbred rats display variable preferences for bittersweet solutions, expressed as preference or avoidance of high concentrations of artificial sweeteners over water. This may reflect individual differences in appetitive/aversive conflict processing that may have predictive validity for disorders of motivation. Here we use a homecage two-bottle choice procedure to examine the test/retest stability and between-tastant consistency in sucralose preference to determine the reliability of bittersweet taste preference. Sucralose is a non-caloric artificial sweetener that is preferred by some rats and avoided by others. We sought to determine whether sucralose preference is consistent with preference of sucrose/quinine solutions that have known sweet and bitter taste qualities, respectively. We give fluid restricted rats 45-minutes homecage access to water and ascending concentrations of sucralose (SUCRA; 0.0025-10mM) or a compound solution of sucrose (116mM) + quinine (0.002-2mM) (SQ). We use a within-subject counterbalanced design (SUCRA or SQ testing) to determine preference of each bittersweet solution relative to water. We observed individual variability in preference for SUCRA and SQ, such that some rats preferred bittersweet solutions over water (preferring) while other rats preferred water over bittersweet solutions (avoiding). Within tastant, this preference remained stable across repeated testing. Between solutions, SUCRA preference scores correlated with SQ scores, suggesting consistent taste conflict processing for both bittersweet solutions. Population level analyses confirmed that preference generalizes across bittersweet solutions, and that rats’ preferences for bittersweet solutions relative to water are stable over time. The test/retest and between-tastant reliability of this taste conflict screening procedure support the potential utility of this model for exploring individual variability in appetitive/aversive conflict processes mediating motivated behavior.

## Introduction

Substance use disorder (SUD) develops only in a subset of individuals that engage in recreational drug use. Approximately 15–30% percent of individuals that try drugs of abuse transition to addiction [1]. Five of eleven DSM V criteria for diagnosing SUD relate to continued drug seeking and taking despite negative consequences [2]. These criteria describe a conflict process, in which motivation for the appetitive drug reward overcomes its potentially aversive consequences. Yet it is unclear whether behavioral responses to appetitive/aversive conflict are pre-existing or drug-induced. There is a need to develop procedures that can capture individual variability in conflict processing prior to drug experience to determine whether this behavioral feature predicts addiction vulnerability. Selectively bred rats that show differences in consumption of sweet taste stimulus, saccharin, have had tremendous utility for predicting many facets of addiction related behaviors across drug classes [3]. Here, we test the reliability of a taste-conflict assay in which outbred rats show individual differences in preference for bittersweet taste stimuli relative to water [4]. Testing trait stability over time and consistency between stimuli is a critical first step for determining whether the taste conflict assay has utility for exploring individual variability in appetitive/aversive conflict processes in outbred rats.

Artificial sweeteners have both bitter and sweet taste qualities at high concentrations [5, 6]. Human studies examining perception of concentration-dependent changes in the taste profile of artificial sweeteners have motivated rodent studies investigating sensory and hedonic taste qualities of artificial sweeteners [3, 4, 7–9]. These sweeteners are perceived as both bitter and sweet in humans, and the behavioral profile of responses in rodents is consistent with the assumption that rodents perceive bitter and sweet qualities of artificial sweeteners in a similar manner. Several rodent studies have elucidated individual differences in the preference for artificial sweetener, sucralose [4, 8–10]. These studies typically use 24 hour homecage two-bottle choice of sucralose and water, demonstrating that some rats avoid and some rats prefer high concentrations of the artificial sweetener relative to water. Sucralose preferring (SP) and sucralose avoiding (SA) rats show concentration-dependent differences in preference of compound taste solutions of quinine and sucrose, which have known bitter and sweet taste qualities, respectively [4]. This work suggests bittersweet taste preference generalizes across bittersweet solutions, and may reflect trait differences in preference for bittersweet taste stimuli. However, this possibly has not been rigorously examined using individual and population level analyses to determine the stability and consistency of bittersweet taste preferences using homecage choice tests.

Here we sought to determine whether limited access (<1 hour) homecage two-bottle choice tests would be sufficient to observe bittersweet taste preference trait differences, which we define as having both individual and population level 1) stability of preference over time, and 2) consistency between SUCRA and SQ preference. To determine whether sucralose preference reflects a trait difference, we independently determined rats’ sucralose (SUCRA) and sucrose + quinine (SQ) preference using a counterbalanced design of two bottle choice tests between water and ascending concentrations of the two bittersweet solutions. We retested rats at isohedonic concentrations to determine whether their individual preferences for each solution remained stable over time. We used within-subject and population level analyses to determine 1) stability of preference over time and 2) consistency between SUCRA and SQ preference.

## Methods

### Animals and housing

Male Long-Evans rats (Charles River Laboratories, Wilmington, MA; n = 36) weighing 330-420g at experiment onset were maintained on a 12:12 h light-dark cycle with light onset occurring at 0630 AM. Two separate cohorts of rats (n = 16 and n = 20) were run using identical procedures described below. There were no differences between cohorts for SQ (F(1,34) = 0.196, p = 0.661) or SUCRA (F(1,34) = .005, p = 0.903) consumption so data were combined. All rats had *ad libidum* access to standard laboratory chow (Tekland, 18% protein, 5% fat, 5% fiber) throughout the course of the experiment. After acclimation to the facility, rats were single-housed and placed on a 23-hour water restriction protocol. All procedures were performed in accordance with the “Guide for the care and use of laboratory animals” (8th edition, 2011, US National Research Council). Animal usage and experimental protocols were approved by the University of Maryland, School of Medicine Institutional Animal Care and Use Committee (IACUC).

We habituated rats to two-bottle choice conditions by providing homecage access to two identical water bottles for 45 minutes each day for a total of nine days. All subsequent preference testing occurred in the home-cage and followed this water restriction and fluid administration protocol.

### Bittersweet solutions

We used two bittersweet solutions, including compound sucrose and quinine (SQ) solution and sucralose (SUCRA). For SQ, we added Quinine hydrochloride dihyrdate (Sigma) at concentrations of 0.002, 0.03, 0.13, 0.25, 0.5, 1, 2 mM to a 4% sucrose (Invitrogen) in purified water (Nestle *Pure Life)* solution. For sucralose we added sucralose (Sigma) at concentrations of 0.0025, 0.025, 0.25, 1.25, 2.5, 5, 10 mM to purified water (Nestle *Pure Life)* (Table 1). We selected these concentrations based on previous work examining bittersweet solution preference [4, 8, 10]. We prepared all solutions immediately prior to testing.

**Table 1:** Tastant concentrations.

Session	1	2	3	4	5	6	7
Sucralose (mM)	0.0025	0.025	0.25	1.25	2.5	5	10
Quinine (mM) in 4% sucrose	0.002	0.03	0.13	0.25	0.5	1	2

### Homecage two-bottle choice test

In 14 homecage test sessions, we gave water restricted rats 45 minutes homecage access to two bottles; one containing SQ or SUCRA and the other containing purified water. We presented the solutions in order of ascending concentration. We reduced location bias by giving each concentration twice, in two consecutive daily sessions that reversed the position of the bittersweet solution. We calculated individual preference scores by dividing the amount of bittersweet solution consumed by the total volume of fluid consumed during the test session (mL bittersweet solution/ (mL bittersweet solution + mL water)). To have a single preference score for each concentration, we averaged preference scores from the two consecutive daily sessions at each concentration. We determined preferring and avoiding groups by averaging preference scores at the 5^th^ and 6^th^ concentrations for each solution (SQ 0.5 mM and 1 mM, SUCRA 2.5 mM and 5 mM), which were based on prior studies examining bittersweet preference [11]. An individual rat was defined as preferring or avoiding independently for SQ and SUCRA. Preferring composite preference scores ranged from 0.60 to 1, while avoiding preference scores ranged from 0 to 0.40. Intermediates had a preference between 0.41 to 0.59.

**Fig 1:**
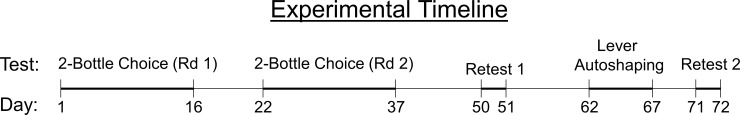
Experimental timeline. We gave two rounds (Rd 1 and Rd 2) of homecage preference testing, counterbalanced for order of bittersweet solution exposure. In round 1 of homecage preference testing, we gave half of the rats (n = 18) two bottle choice of water and ascending concentrations of SQ, while we gave the other half of the rats (n = 18) two bottle choice of water and ascending concentrations of SUCRA. In round 2, we gave rats two bottle choice of water and ascending concentrations of the alternate solution that they had not experienced in round 1. After these two rounds, we gave rats two retest sessions to determine whether their preference for the round 1 and round 2 solutions remained stable. For the Retest 1 sessions, we gave rats two bottle choice of water and C5 and C6 (counterbalanced) of the round 1 solutions, and for Retest 2, we gave rats two bottle choice of water and C5 and C6 (counterbalanced) of the round 2 solutions. Rats were tested in Pavlovian lever autoshaping between Retest 1 and Retest 2 (data not presented).

The experimental timeline is presented in Fig 1. We gave two rounds of homecage preference testing, counterbalanced for order of bittersweet solution exposure. In round 1 of homecage preference testing, we gave half of the rats (n = 18) two bottle choice of water and ascending concentrations of SQ, while we gave the other half of the rats (n = 18) two bottle choice of water and ascending concentrations of SUCRA. In round 2 of homecage preference testing, we gave rats two bottle choice of water and ascending concentrations of the alternate solution that they had not experienced in round 1. After these two rounds, we gave rats two retest sessions to determine whether their preference for the round 1 solution remained stable. During these two retest sessions, we gave rats two bottle choice of water and C5 and C6 (counterbalanced) of the round 1 solution. The retest preference scores were calculated in the same manner as the preference score during initial testing. In addition, we retested rats to determine whether their preference for the round 2 solution remained stable. Both retest rounds occurred between 24–44 days after the last exposure to that tastant. Rats received five sessions of Pavlovian lever autoshaping as previously reported [12] (between the 1^st^ and 2^nd^ retest, but data from this phase is not presented here. At the end of the experiment rats were deeply anesthetized with isoflurane (~90 s) and perfused transcardially with 100 ml of 0.1 M PBS with 1.25% sodium nitrite, followed by 400 ml of 4% para-formaldehyde in 0.1 M sodium phosphate, pH 7.4.

### Statistical analyses

We analyzed homecage two bottle choice data using the SPSS statistical software (IBM) by repeated measures ANOVAs, and significant main effects and interaction effects (p < 0.05) were followed by Bonferroni post-hoc tests. We used a McNemar chi square analysis using SPSS software to evaluate between-tastant shifts in preference phenotype. We performed regression analyses of test-retest preference scores and between tastant preference scores using Prism (Graphpad). To evaluate shifts in population distribution, we employed Wilcoxon Sign-rank tests in Matlab (Mathworks). We describe dependent measures and factors used in the statistical analyses in the results section below.

## Results

**Fig 2:**
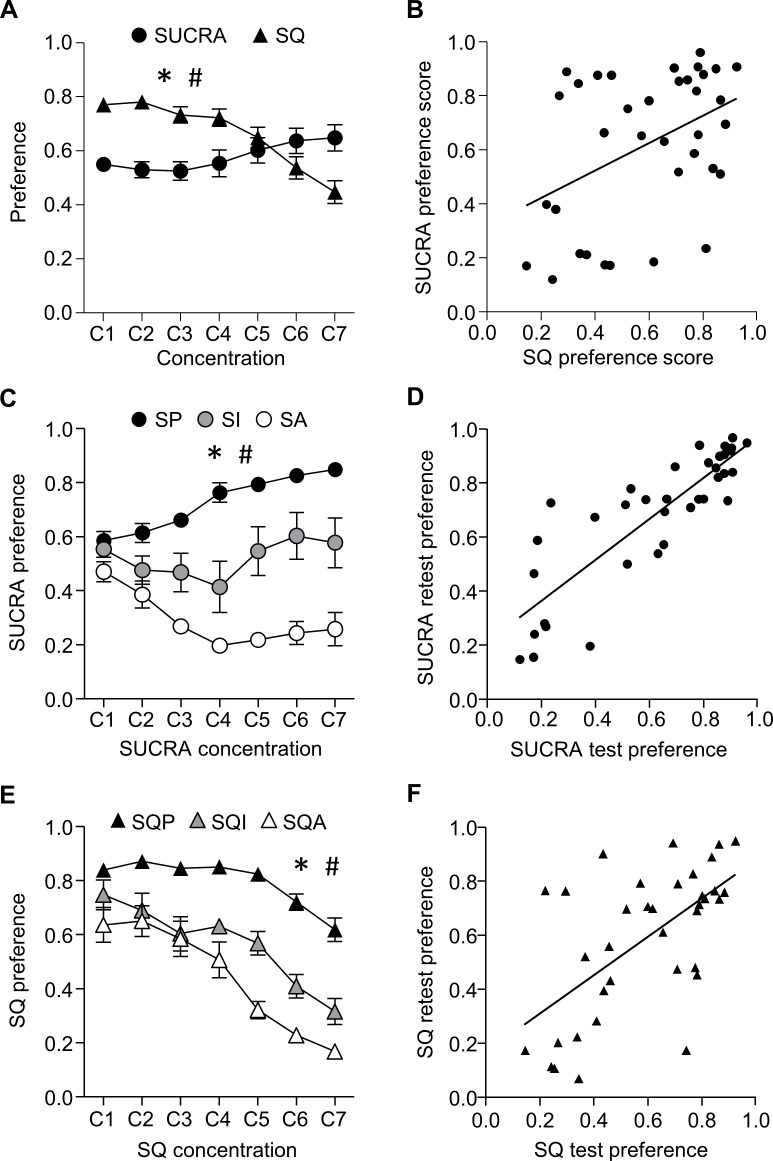
Stability and consistency in preference for isohedonic concentrations of SUCRA and SQ. Data are mean +/- standard error of the mean (SEM). Preference score = mL tastant solution/ (mL tastant solution + mL water) for each concentration. A. Concentration dependent preference of SUCRA and SQ relative to water for all rats. B. SUCRA and SQ preference scores at isohedonic (5^th^ and 6^th^) concentrations are correlated. C. SUCRA preference scores plotted for Sucralose Preferring (SP), Sucralose Avoiding (SA), and Sucralose Intermediate (SI) rats for all SUCRA concentrations tested. SP, SA, SI groups were determined by calculating each rat’s preference for SUCRA at isohedonic concentrations. SUCRA preference scores for SP and SA groups were significantly different at the four highest concentrations tested. D. SUCRA preference scores at first test and retest are correlated. E. SQ preference scores plotted for SQ Preferring (SQP), SQ Avoiding (SQA), and SQ Intermediate (SQI) for all SQ concentrations tested. SQ preference scores for SQP and SQA groups were significantly different at the three highest concentrations tested. F. SQ Preference scores at initial test and retest are correlated. *Significant main effect of Concentration; # significant Concentration × Tastant (A) or Concentration × Preference interaction (C, E).

In order to explore the generalizability in preference for different bittersweet solutions, we gave rats one round of SUCRA and one round of SQ preference testing, with order of exposure counterbalanced. We analyzed the within-subject data to determine the concentrations at which SUCRA and SQ are each similarly preferred to water, which we refer to as the isohedonic concentrations. To find these isohedonic concentrations, we examined how preference for each bittersweet solution over water changed with increasing concentrations of SUCRA and SQ. We analyzed the data using a repeated measures ANOVA with the within subject factors Tastant (SUCRA, SQ) and Concentration (1–7). There were main effects of Tastant (F_1,210_ = 7.097, p = 0.012) and Concentration (F_6,210_ = 7.241, p<0.0001), and a Tastant x Concentration interaction (F_6,210_ = 32.690, p<0.0001; Fig 2A). Post-hoc t-tests revealed that SQ was more preferable to water than was SUCRA to water at concentrations 1–4, (p’s <0.0008), but the two were similarly preferred to water at the 5^th^ concentration (p>0.05). Rats had a preference for SUCRA to water over SQ to water at the 6^th^ and 7^th^ concentrations (p’s<0.05). Additionally, we found that preference scores for both solutions were correlated when the 5^th^ and 6^th^ concentrations for each solution are averaged (r^2^ = 0.18, p = 0.0099; Fig 2B). These data suggest that on individual level, there is consistency across bittersweet solutions at the isohedoinc (5^th^ and 6^th^) concentrations.

We next examined individual differences in SUCRA and SQ preference across the ascending concentrations of the two bittersweet solutions. Using the preference criterion described in the methods section, we characterized the rats as SUCRA preferring (SP, n = 22), SUCRA avoiding (SA, n = 10), or intermediates (INT, n = 4) using preference scores at the isohedonic concentrations. We analyzed SUCRA preference scores for all seven concentrations using a mixed ANOVA with between subject factor of Preference and within subject factor of Concentration (Fig 2C). There were main effects of Preference (F_2,33_ = 122.184, p<0.0001), Concentration (F_6,198_ = 5.252, p<0.0001) and a Preference by Concentration interaction (F_12,198_ = 13.839, p<0.0001). Post-hoc tests revealed that preference scores for SP rats and SA rats were significantly different from concentrations C2-C7 (0.025–5 mM sucralose; p’s<0.0008, corrected for multiple comparisons). Preference scores for the isohedonic concentrations of SUCRA were stable across repeated testing, with correlated preference scores at first test and retest 24–44 days later (r^2^ = 0.73, p<0.0001; Fig 2D).

Rats also showed individual differences in preference for ascending concentrations of SQ versus water (Fig 2E). Using the preference criterion described in the methods section, we characterized rats as SQ Preferring (SQP, n = 20), SQ Avoiding (SQA, n = 9), or INT (INT, n = 7). We analyzed the SQ preference data using a mixed ANOVA with between subject factor of Preference and within subject factor of Concentration. There were main effects of Preference (F_2,33_ = 66.570, p<0.0001) and Concentration (F_6.198_ = 60.354, p<0.0001), and a Preference by Concentration interaction (F_12,198_ = 5.698, p<0.0001). Post-hoc tests revealed that preference scores for SQP rats and SQA rats were significantly different at all concentrations tested (p’s<0.0005, corrected for multiple comparisons). Preference scores for the isohedonic concentrations of SQ were stable across repeated testing, with correlated preference scores at first test and retest 24–44 days later (r^2^ = 0.37, p<0.0001; Fig 2F). Thus, for both SUCRA and SQ, rats show stable preferences across repeated testing.

**Fig 3:**
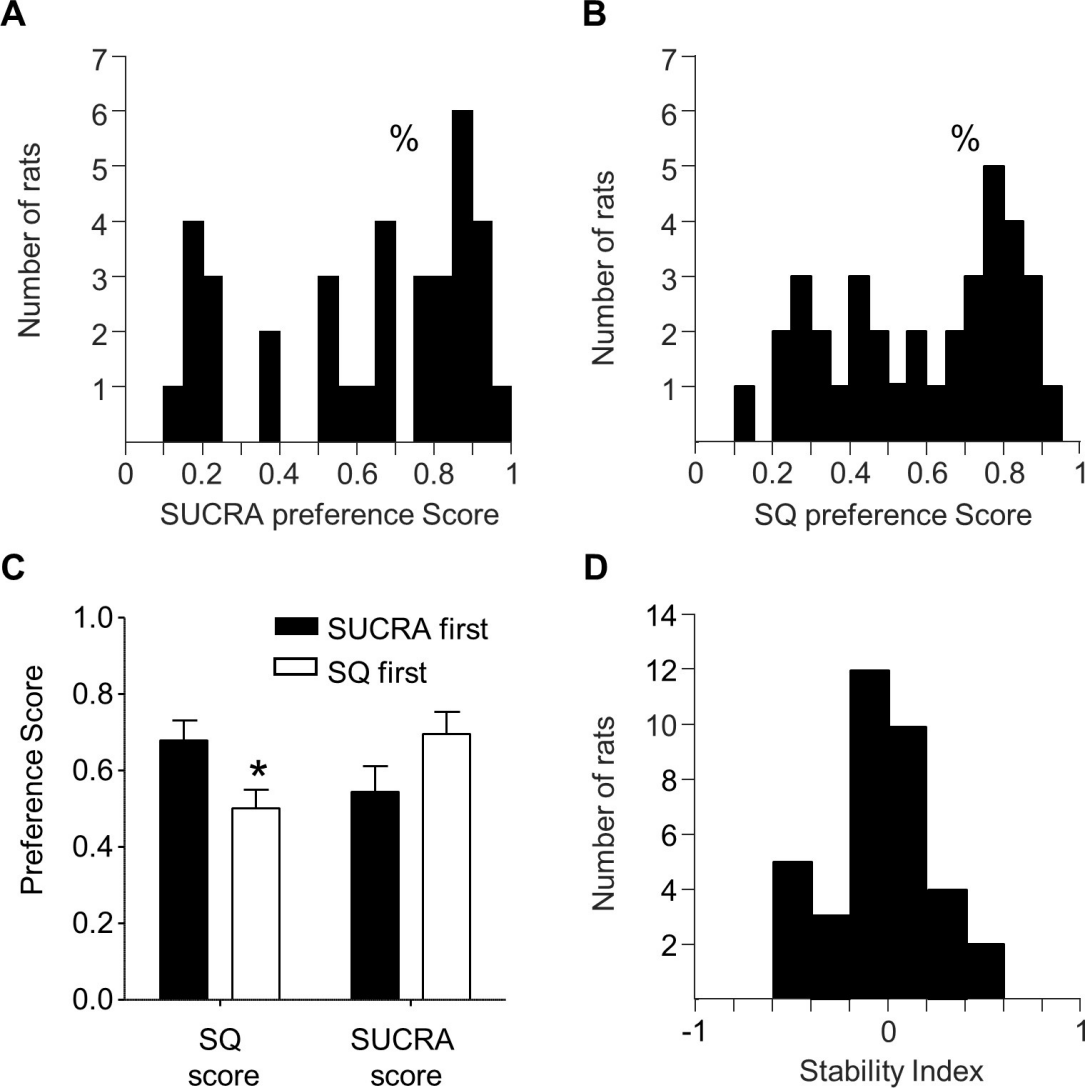
Population level consistency in SUCRA and SQ preference. A. Population distribution of SQ Scores are significantly shifted rightward, indicating that rats overall preferred SQ. B. Population distribution of SUCRA Scores are significantly shifted rightward, suggesting that the population overall preferred SUCRA. %significant shift in population. C. Across the entire population, SQ preference scores were higher when rats were exposed to SUCRA first relative to SQ first. D. Population distribution of the Stability Index (SI) score, which is calculated by subtracting each rats’ SUCRA preference score from their SQ preference score. The distribution is not significantly shifted from zero. *significant post-hoc t-test between SUCRA first and SQ first.

We also performed population level analyses on preference scores for the isohedonic concentrations (5^th^ and 6^th^ averaged) for each solution (Fig 3A and 3B). Using a Wilcoxon sign-rank test, we observed no difference in SUCRA and SQ preference score population distributions (Z = -0.50, p = .6151), demonstrating consistency on a population level between the two bittersweet solutions. Wilcoxon sign-rank tests also revealed that both SUCRA and SQ preference score distributions were significantly shifted above zero (Z = 2.545, p = .011; Z = 2.310, p = .021, respectively), indicating the majority of rats prefer both bittersweet solutions over water.

Next, we used a McNemar Chi-Square to examine consistency in preference group (Prefer, Avoid) across tastant solutions (SUCRA, SQ) for the entire population. In order to conduct the McNemar Chi-Square test we classified rats (n = 36) as Preferring if preference scores were above 0.5 and Avoiding preference scores were below 0.5 (Table 2). Using these criteria, the majority of rats (n = 28) showed a consistent preference between tastants, such that 20 preferred both solutions and eight rats avoided both solutions. Only eight rats displayed inconsistent preferences between solutions, and while the sample size is low, the McNemar test confirmed the inconsistency in preference between tastants was likely due to chance (χ^2^ (1, n = 36) = 1.125, p = .144).

**Table 2:** Consistency of preferring/avoiding across tastant solutions.

	SUCRA Preferring	SUCRA Avoiding
SQ Preferring	20	2
SQ Avoiding	6	8

Because of this low sample size, we addressed the possibility that the inconsistency we observe across tastant solutions is due to the order of tastant exposure. We performed repeated measures ANOVAs on SQ and SUCRA preference scores, with between-subjects factor of First Tastant (SQ first, SUCRA first) and within-subjects factor of Tastant Concentration (1–7). There were no main effects of First Tastant on either SQ or SUCRA scores (SQ first (F(1,34) = 3.11, p = .08), SUCRA first (F(1,34) = 3.44, p = .07)). However, we do observe a First Tastant x SQ Concentration interaction (F(1,34) = 5.45, P < 0.01). Post-hoc t-test reveals SQ preference scores were higher when we exposed rats to SUCRA before SQ solutions (Fig 3C). Notably, this was not the case for SUCRA preference scores (First Tastant x SUCRA Concentration; F(1,34) = 2.05, p = 0.129, Fig 3C). This suggests that SUCRA preference, while correlated with SQ preference, is more stable than SQ preference, the latter of which may be sensitive to experience-dependent factors like order of exposure.

Also at the population level, we generated a Stability Index (SI) score, which we calculated by subtracting each rats’ SUCRA preference score from their SQ preference score. Thus, a Stability Index score of zero indicates rats with consistent preference scores for both SUCRA and SQ, while negative SI scores indicate rats that preferred SQ but avoided SUCRA, and positive SI scores indicating rats that preferred SUCRA but avoided SQ. The distribution of Stability Index scores is shown in Fig 3D. We analyzed the population distribution using a Wilcoxon sign-rank test which showed the SI distribution was not significantly shifted above/below zero (Z = -0.54, p = 0.58). This analysis demonstrates that on a population level, rats’ preference for two different bittersweet solutions was consistent. Taken together, the population analyses suggest that rats’ preferences for bittersweet solutions relative to water are stable within and between tastants. This suggests individual differences in bittersweet taste preference reflect a continuum of stable behavioral phenotypes that may have utility for studying individual differences in appetitive/aversive conflict processing.

## Discussion

Here, we sought to determine whether outbread rats show individual differences in homecage sucralose preference that are 1) stable over time, and 2) consistent with preference of a known bittersweet compound solution of sucrose + quinine. We found that individual rats’ preferences for each solution remained stable over time and with intervening experience with the other bittersweet solution. Similar to previous studies, we observed a range of preference scores at the isohedonic concentrations, demonstrating that individual rats vary along the bittersweet preference/avoidance continuum [4, 8, 9]. We found that at isohedonic concentrations, sucralose preference correlated with preference of the sucrose/quinine solution. Despite classifying individual preference based on consumption at the isohedoinc concentrations, concentration-dependent choice of bittersweet solutions relative to water was significantly different between preferring and avoiding groups at every concentration tested. We observed overlapping population distributions for sucralose and sucrose/quinine preference scores, which were significantly right-shifted towards preferring for both solutions. Across the population, a majority of rats showed stability in preferences between solutions. While some rats displayed inconsistencies in preference within or between taste solutions, the number of rats shifting in either direction along the continuum was below what would be expected by chance, however order of tastant exposure may have contributed to the observed inconsistencies. Together, our results suggest that sucralose preference generalizes to preference of a known bittersweet compound solution of sucrose and quinine and those preferences for both bittersweet solutions remain stable over time and experience.

Prior work has shown that the concentration-dependent preference for sucrose/quinine solutions is different for sucralose preferring and avoiding rats [4, 10]. Similar to the present study, rats in a previous study [4] were tested with ascending concentrations of quinine in a fixed sucrose concentration. Compared to sucralose preferring rats, sucralose avoiding rats showed an accelerated reduction in preference of sucrose/quinine solutions as a function of increasing quinine concentration. This group difference was interpreted as stable preference across bittersweet solutions. Our study extends these findings showing that individual rats’ SUCRA and SQ preference scores are correlated, and that the population distributions at the isohedonic concentrations for these two solutions are overlapping. Furthermore, our study demonstrates that overwhelmingly rats display consistent preferences for each solution across repeated testing. This additional information provides much needed assessment of trait stability that would be necessary for using the sucralose preference as a model to investigate individual differences in other motivated behaviors.

The proportion of sucralose preferring rats observed here is similar to the first study exploring individual variability in sucralose preference [9]. However, the proportion of sucralose avoiding rats in the present study contrasts with subsequent studies that observed a majority of rats avoid sucralose [4, 8]. There are several methodological factors that differ between our study and prior reports that may account for this difference. First, prior studies used 24 hour homecage access 2 bottle choice testing, measuring fluid consumption over a greater time period than in the present study using 45-minute access to two bottle choice. In the 24 hour access conditions of prior sucralose preference studies, rats were not fluid restricted to determine preference groups [4, 8, 10]. Notably, these studies reported that 24 h homecage preference under *ad libitum* fluid conditions consistently predicted brief access licking and consumption of bittersweet solutions under acutely fluid restricted conditions [10]. In contrast, we chronically fluid restricted rats so that we could test preference in limited access sessions. Thus, in the present study, rats’ consistently high motivational state to consume fluids may have biased the population towards bittersweet preference. Other factors that are less likely to play a role in the different sucralose preference distributions observed across studies include rat strain, sex and estrous cycle phase. Each of these factors have been systematically evaluated and shown to have little influence over sucralose preference distributions [8, 13].

We observed some inconsistencies between SUCRA and SQ preferences. Most notably, we observed that order of tastant exposure affected SQ preference scores. This likely contributed to the inconsistency between tastant solutions. Other considerations with regard to the influence of taste on inconsistent preferences, the pattern of preference scores across concentrations differs between taste stimuli. Across the population, low concentrations of SQ were consistently preferred over water with decreasing consumption as quinine concentrations increased in all groups. However, the lowest concentration of SUCRA was similarly preferred to water, with SP rats increasing and SA rats decreasing consumption of SUCRA as concentration increased. These patterns suggest sweet perception dominance for low concentrations of SQ, but indifference at low concentrations of SUCRA. Thus, inconsistencies in preferences between taste stimuli could be attributable to negative/positive contrast within a specific concentration series. Notably, differences in caloric value and osmolality between SQ and SUCRA at the isohedonic concentrations may also affect preference in two-bottle choice. Rats may consume more sucrose-quinine relative to water for the caloric value of sucrose, while sucralose has no caloric value and thus consumption would not be influenced by this factor. Rats may also consume more water during choice tests with higher osmolality solutions, which may drive homeostatic water consumption to neutralize potential electrolyte imbalance. In limited access 2-bottle choice, this may influence preference scores. Both caloric value and osmolatity differences between taste solutions could contribute to the inconsistencies in preference that we observe in the population at isohedonic concentrations.

A number of factors suggest SUCRA preference has greater utility than SQ preference for assessing risk for conflict processing and disorders of motivation. First, SUCRA preference shows stability without being influenced by order of tastant exposure, which is not the case for SQ preference. The more pronounced bimodality of SUCRA distributions relative to SQ distributions and the fewer number of inconsistent SUCRA avoiders relative to SQ avoiders also suggest that preference of non-nutritive, sucralose, may have more utility as a screening procedure than SQ preference. The extent to which bimodality of the SUCRA distribution reflects biological differences between SP and SA rats, this preference phenotype may be better suited to predict variation in conflict processing, which we postulate has common neurobiological underpinnings. However, the utility of SQ screening may be improved by examining the stability and consistency of taste preference for compound SQ mixtures when quinine concentrations remain fixed and sucrose concentrations vary.

Several studies have speculated and even designed studies to directly examine the nature of these individual differences in preference for bittersweet solutions [3, 4, 8–11]. Individual differences could arise from genetic differences in taste perception. Notably, the aversive bitter or metallic taste qualities of high concentrations of artificial sweeteners result from activation of bitter taste receptors (T2R) and/or transient receptor potential vanilloid-1 (TRPV1) receptors [14–16]. Genetic mutations in either one of these receptors may contribute to differences in sensory processing of sucralose and other bittersweet solutions. There is evidence that SP and SA rats show differences in sucralose perception, with SP rats classifying high concentrations of sucralose as having “sucrose-like” qualities, whereas SA rats don’t [10]. Having controlled for motivation in that study, individual differences in sucralose acceptance were explained by perceptual variation in taste quality, and likely reflect differences at the level of taste perception. However, in the same study, differences in the acceptance of sucrose-quinine mixtures were not predicted by perceptual variation in taste quality. Our study and others [4] have identified a relationship between sucralose acceptance and sucrose/quinine acceptance, suggesting consistency in preference and avoidance across solutions may arise from differences in higher-order taste processing in gustatory brain regions and downstream hedonic processing in reward circuitry. Consistent with this idea, while increased palatability of sweet solutions is typically associated with increased number of lick bouts and increased lick rates [17], a microstructural lick analysis study demonstrated that sucralose preferring rats have consistent lick bouts and reduced lick rates with increasing sucralose concentrations [11]. Reduced lick rates are associated with aversive taste qualities [17]. Thus, the bitter taste detection in SP and SA may not drive differences in consumption; instead preferring rats may be more motivated to consume high concentrations of sucralose than sucralose avoiding rats, perhaps due to enhanced motivational salience of sweet stimuli [10, 11].

Rats selectively bred for high and low consumption of sweet taste stimulus, saccharin, have had tremendous utility for predicting many facets of addiction-related behaviors across drug classes [3]. Rats that consume high quantities of saccharin (HiS) show consistently greater vulnerability across drug classes than rats that consume low quantities of saccharin (LoS) [3]. Studies using HiS and LoS inbred lines have also examined artificial sweetener consumption, demonstrating LoS rats consume less sucralose and other artificial sweeteners than HiS rats [13, 18–20]. While consumption of bitter tastes alone does not differ between LoS and HiS inbred rats, LoS rats avoid sucrose/quinine mixtures at lower quinine concentrations than HiS rats [19], similar to what has been found with outbred Sucralose Avoiding relative to Sucralose Preferring rats [4]. The Sucralose Preferring and Avoiding phenotypes may have similar utility for predicting addiction related behaviors in outbred rats.

The enhanced motivational salience of appetitive relative to aversive stimuli has relevance for substance use disorder (SUD). Several criteria for diagnosing SUD describe a conflict process, in which motivation for the appetitive drug reward overcomes its potentially aversive consequences [2]. The current study validates the reproducibility and the test/retest reliability of sucralose preference procedures. Our limited access procedures adequately capture behavioral variability for appetitive/aversive taste preference, which is stable over time, consistent across taste stimuli and is evident prior to drug-experience in outbred rats. Future studies are warranted to explore the potential utility of this model for predicting individual differences in natural and drug reward seeking under conflict conditions.

## Supporting information

S1 Data PLOSone Data.xlsx shares raw data for each figure presented in the manuscript. (XLSX)
